# Brownlow Hill Poor Law Infirmary

**Published:** 1910-01-15

**Authors:** 


### Editor's Letter-Box.

### BROWNLOW HILL POOR LAW INFIRMARY.

*To the Editor of* The Hospital.

Sir,—I have visited the above hospital on several occasions recently; and, notwithstanding the attempted explanations of the Select Vestry at their last committee meeting, I can, as the result of close observation and full inquiries, confirm the main charges made against the institution by Sir H. Burdett in your columns. It will be observed that the Vestry did not contradict the charges made, but contented themselves with general abuse of Sir H. Burdett, with which was thrown in some misleading statements that in no way met the case made against them.

The statement that there is no proper classification of the persons entering the maternity wards on admission is correct. "Unfortunates," some having a loathsome disease and some not, are admitted for shelter and treatment to the maternity wards, and beside them are placed wives of poor workmen, perhaps ill or out of work, and who are unable to afford proper accommodation for their wives at this critical period; also perhaps a young girl who has for once lapsed from the path of virtue. All are thrown together. Surely better than this is possible. Why not provide a small ward for the first-mentioned class? An attempt was made by the quotation of mortality amongst maternity patients to show splendid results for Brownlow Hill, but if actual comparison were made with ordinary maternity institutions it would be found that the figures quoted by a lady Guardian would not bear examination. At general maternity hospitals all deaths from every cause are counted, but that course is not observed at Brownlow Hill, where deaths from purely maternity causes only are counted. The baths and lavatories are about as bad as they can be, and no description could paint them worse than they are. The former are cased with wood and painted. The wood is worn, and the paint is absent in many places. When it is recollected that the baths are also used as washtubs for cleansing mackintosh sheets as required, their sanitary condition can be readily imagined. The lavatories are no better. The Guardians do not defend; but, on the other hand, they propose no improvement of either baths or lavatories. Practically Sir H. Burdett's description is admitted; fibre beds and flock pillows at the present day are unknown in general hospitals. The Guardians should recollect that in the case of a large portion of their patients, the working man or his wife who is unable to obtain admission to a general hospital in Liverpool is forced into Brownlow Hill. At the former the main endeavour of the medical staff is to do everything that is possible to put the patient on his or her legs again at the earliest moment. By so doing the expense at the hospital is closed without delay, and the patient is restored to the army of workers. At Brownlow Hill, owing to the lay rule of Guardians and lay officials, the proper medical treatment of the patient is lost sight of, a longer stay in the hospital is thus rendered necessary, and the "army of workers" in the meantime deprived of the services of the persons improperly held up by the Guardians owing to improper treatment.

A lady Guardian at the meeting referred to stated that she had a fibre bed sent to her residence, and "slept on it two or three nights." The bed was, no doubt, a new one, not the worst that could be found, but it is not stated that a flock pillow, new or old, was used by the lady. I ask, How would she like, if laid by with pneumonia or typhoid or other serious illness, to use an old fibre bed and a well-worn flock pillow? She might be prepared to do so, but where would she get a doctor who would sanction such a freak on her part? At Brownlow Hill the fibre bed and flock pillow are considered by the Guardians as proper for all classes of hospital patients. If a medical superintendent were in sole charge of the hospital I wonder what would be his opinion?

So much for the questions raised by Sir H. Burdett, but he missed a most important matter, whether by accident or design, on the part of those who accompanied him through the hospital, I cannot say. The operating theatres, male and female, are alike. I shall describe one, it will do for both, although one is more than a dozen years in existence, and the other about half that period. The pattern of the first was so excellent that departure from it when the second was erected was not thought of. The theatres (both) are situated on the top floor, and the lifts available where necessary. Patients removed to and from the wards to the theatre are conveyed, up and down stairs with stone steps, in a sling, either by convalescent patients or inmates of the union, after operation frequently under an anæsthetic. There is no preparation room attached to the theatre. When operations pass beyond one, the second patient for preparation has to pass into the theatre while the first is under operation, and the second is placed under the influence of the anæsthetic with a small screen between the patients and within earshot of all that is passing at the other table. It may well be asked, can such a thing be conceived as existing in enlightened Liverpool in the twentieth century. The operating tables are quite obsolete of the gridiron pattern, with no mechanism for adjusting and without means for heating. The roof of the theatre is uneven, the electric lights are cumbersome, and both are well adapted for holding dust and quite opposed to up-to-date ideas of a properly constructed theatre of to-day. There is no occasion to push the point that sterilisation work carried out in such a place cannot be regarded as satisfactory. It is a pity that Sir H. Burdett did not obtain a glance at one of the theatres. His description would have been more forcible than mine. Such, however, are the theatres provided for the poor who enter Brownlow Hill for surgical treatment. Some of the Guardians, at least, are aware of the condition of affairs described, but it is probable that they are afraid to touch the tottering fabric in view of the serious consequences that might follow. Their reputation as excellent Guardians might suffer, but in view of Sir H. Burdett's exposures how stands their reputation now?

It is stated by the local press here that a Local Government Board inquiry is shortly to be held. If so, to be effective an inquiry should be first held by a medical inspector of the Board. After that, a full inquiry should be made into the hospital administration at Brownlow Hill, and the Guardians should be forced to bring the present inefficient system up to an efficient one—gradually, of course, as large cost at once is a question of importance; but the work should be entered upon without delay and pushed on until all abuses are remedied.

Faithfully yours,
An Onlooker.

